# Beyond Composition: Structure–Activity Relationships in Bioactive Deep Eutectic Systems

**DOI:** 10.3390/pharmaceutics18080990

**Published:** 2026-08-11

**Authors:** Paulina Hernández, Catherine Klein, Paola R. Campodónico, Belén Olivares

**Affiliations:** Centro de Química Medica, Facultad de Medicina, Clínica Alemana-Universidad del Desarrollo, Santiago 7690000, Chile; paulina.hernandez@udd.cl (P.H.); ckleinr@udd.cl (C.K.); pcampodonico@udd.cl (P.R.C.)

**Keywords:** deep eutectic systems, structure–activity relationships, supramolecular organization, physicochemical microenvironment, hydrogen-bond networks

## Abstract

Deep eutectic systems (DESs) have evolved from sustainable solvent alternatives to promising bioactive platforms with reported antimicrobial, anti-inflammatory, regenerative, cryoprotective, and cytoprotective properties. However, despite the growing number of biological studies, the mechanistic basis of these effects remains poorly understood because biological activity is still interpreted predominantly from the chemical identity of the hydrogen-bond donor and acceptor, rather than from the supramolecular organization of the eutectic system itself. This review is intended to provide anyone interested in the biomedical and pharmaceutical applications of DESs with a conceptual framework for understanding how supramolecular organization may influence the biological performance of DES-based systems, without requiring extensive expertise in physical chemistry. It critically analyzes the current evidence linking DES structure with biological function. The literature reveals that many reported biological responses cannot be fully explained by the properties of the individual constituents alone, supporting the existence of emergent physicochemical behavior associated with eutectic formation. Current evidence further demonstrates that DESs are dynamic supramolecular systems characterized by hydrogen-bond networks, nanoscale heterogeneity, hydration-dependent structural rearrangement, and persistent local organization under biologically relevant conditions. These structural features generate localized physicochemical microenvironments capable of modulating membrane organization, protein hydration, osmotic balance, and biomolecular interactions, providing a plausible mechanistic basis for the diverse biological effects reported to date. Our analysis also highlights a fundamental disconnect between the extensive physicochemical characterization of DESs and the predominantly composition-based interpretation of their biological activity. While conventional Quantitative Structure–Activity Relationship (QSAR) approaches rely on molecular descriptors of individual components, they fail to capture the higher levels of organization that characterize these dynamic multicomponent systems. Based on concepts established in supramolecular chemistry, self-assembled biomaterials, colloidal science, and soft matter, we propose a Hierarchical Structure–Activity Relationship (H-SAR) framework in which biological activity emerges from successive levels of organization extending from molecular composition and hydrogen-bond networks to nanostructural organization, hydration-dependent restructuring, localized physicochemical microenvironments, and biological interfaces. This framework provides a mechanistic basis for interpreting DES bioactivity and could offer a conceptual roadmap for the rational design, predictive modeling, and biomedical translation of next-generation bioactive deep eutectic systems.

## 1. Introduction

Deep Eutectic Systems (DESs), commonly referred to as Deep Eutectic Solvents, are multicomponent systems in defined molar ratios that interact through strong intermolecular forces, including hydrogen bonding, electrostatic interactions, van der Waals forces, and, in some cases, hydrophobic interactions. As a result, they share common structural features arising from cooperative intermolecular interactions, leading to physicochemical properties that differ markedly from those of the individual constituents. Their most characteristic and extensively studied emergent property is the pronounced depression of the melting temperature relative to that of the isolated components. Since their formal introduction by Abbott and co-workers, DESs have traditionally been categorized into four main types, according to the chemical nature of their components. Type I DESs are formed by a quaternary ammonium salt and a metal chloride; Type II combine a quaternary ammonium salt with a hydrated metal chloride; Type III, the most extensively studied class for biomedical applications, consist of a hydrogen-bond acceptor (HBA), typically choline chloride or a related quaternary ammonium salt, and a hydrogen-bond donor (HBD), such as urea, organic acids, polyols, sugars, or amides; and Type IV are composed of metal salts, acting as hydrogen-bond acceptors, combined with hydrogen-bond donors [1]. DESs have attracted considerable interest as sustainable alternatives to conventional solvents owing to their low volatility, synthetic simplicity, tunable composition, and broad compatibility with organic and biological systems [2,3,4].

The versatility of DESs extends well beyond their role as solvent replacements. Their highly tunable physicochemical properties have enabled applications across extraction processes, catalysis, electrochemistry, biomass valorization, materials engineering, and pharmaceutical formulation [5,6,7]. In many of these contexts, DESs have been exploited for their ability to enhance the solubility and stability of active compounds, improve processing performance, and modulate formulation characteristics in ways that cannot be readily achieved using conventional solvent systems [8,9].

During the past decade, however, interest in DESs has progressively shifted from their technological utility toward their potential biological functions. This transition has been particularly evident for Natural Deep Eutectic System (NADESs), a subclass of predominantly Type III DESs [10], which are composed of naturally occurring metabolites such as amino acids, sugars, organic acids, and quaternary ammonium compounds such as betaine or choline derivatives. For simplicity, NADESs are referred to as DESs in this text. Increasing evidence indicates that these systems can exert biological effects that frequently exceed, attenuate, or qualitatively differ from those expected from their isolated constituents [11,12].

Accumulating reports demonstrate that DESs can influence biological systems at multiple levels, including membrane integrity, protein stability, oxidative stress regulation, microbial viability, biofilm architecture, and tissue regeneration. Collectively, these observations challenge the traditional view of DESs as passive solvent matrices and instead suggest that they may function as biologically active systems whose properties emerge from collective intermolecular organization.

Despite the rapid expansion of this field, the mechanistic basis of DES bioactivity remains poorly understood. Most studies continue to interpret biological responses through a reductionist compositional framework, attributing observed effects primarily to the intrinsic activity of individual components while largely overlooking the potential contribution of eutectic organization itself. As a result, a substantial conceptual gap persists in our understanding of the structure–activity relationships (SARs) governing DES-mediated biological effects. This challenge differs fundamentally from conventional medicinal chemistry. In classical SAR approaches, biological activity is correlated with well-defined molecular structures; by contrast, DESs represent dynamic physicochemical systems whose functional behavior emerges from collective interactions, including hydrogen-bond (HB) networks, nanoscale heterogeneity, water-mediated restructuring, and adaptive supramolecular organization. Consequently, biological activity may not be dictated solely by chemical composition, but also by the structural hierarchy that develops within the eutectic system.

A major obstacle to advancing this field is the absence of standardized methodologies capable of characterizing DES organization under in vitro experimental conditions that more closely mimic the physicochemical and biological environment encountered in vivo. Although considerable progress has been made in elucidating the molecular and nanoscopic structure of DESs under controlled physicochemical environments, much less is known about how these structural features evolve upon dilution, hydration, interaction with biomolecules, or exposure to complex biological media. This limitation has hindered mechanistic interpretation and restricted the development of predictive frameworks for DES design.

**Figure 1:**
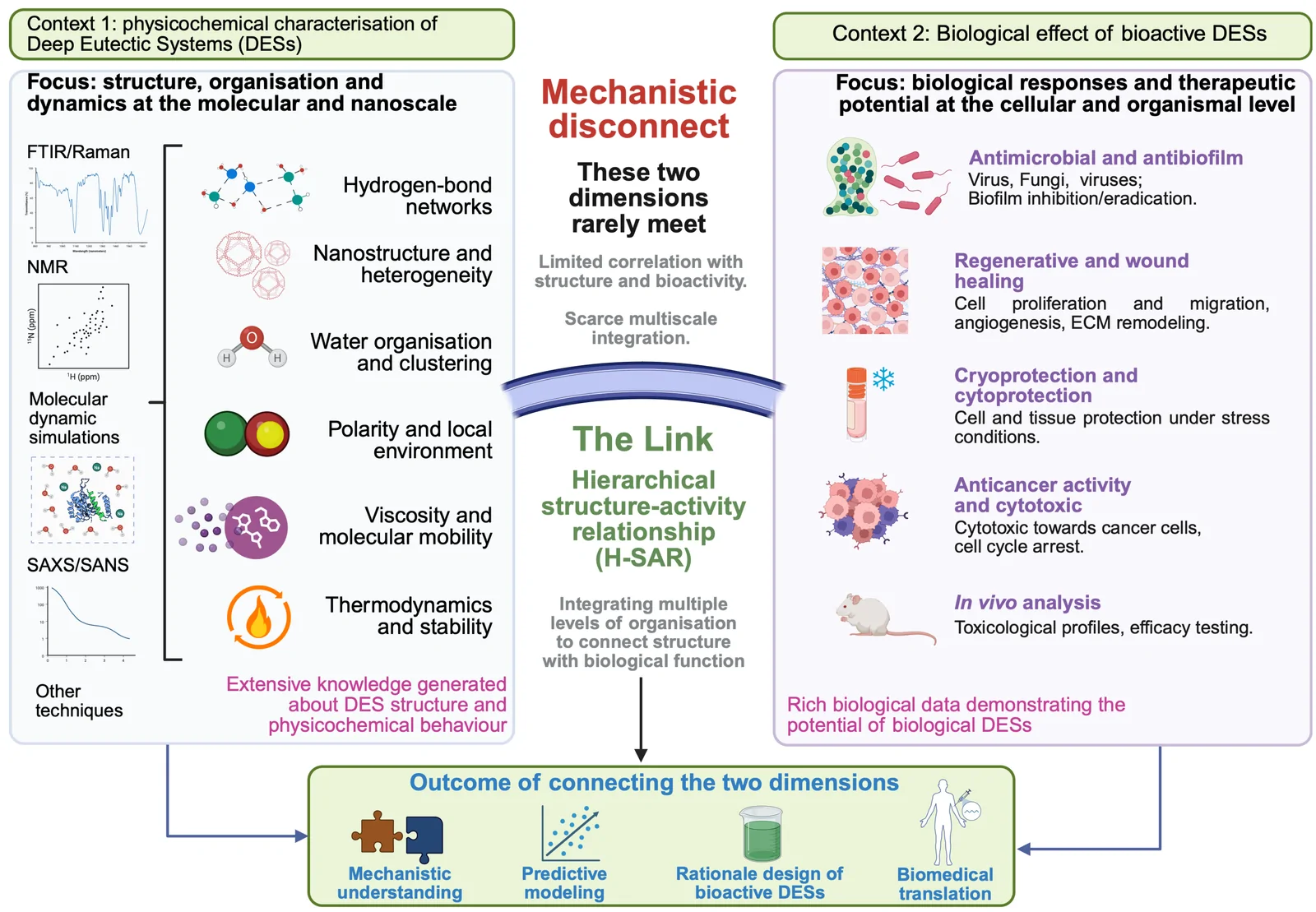
The current mechanistic gap between physicochemical characterization and biological evaluation of Deep Eutectic Systems: a rationale for proposing a Hierarchical Structure–Activity Relationship (H-SAR) framework. Created in BioRender. UDD, D. (2026) https://BioRender.com/zvstllx.

In this review, we examine the missing relationship between DES structure and biological activity (Figure 1). We first summarize the biological effects reported for DES-based systems and subsequently analyze the micro- and nanostructural features that may underpin these responses. We further discuss structural persistence under biologically relevant conditions, the role of DES-mediated microenvironment modulation, and the growing conceptual framework supporting DESs as functional chemical entities. Finally, we propose future directions toward the development of predictive structure–activity relationships capable of transforming DES research from empirical screening into rational design.

### Scope and Literature Search

PubMed and ScienceDirect were searched for English- and Spanish-language records published between January 2016 and May 2026, with earlier foundational works incorporated through reference list screening. Searches were run independently by four authors. Two search strands were applied, both built on a common concept block (“deep eutectic” OR “NADES” OR “low transition temperature mixtures”). The first combined this block with terms for antimicrobial and antibiofilm activity, inflammation and immunomodulation, oxidative stress, cytoprotection and regeneration, cryoprotection, and cytotoxicity. The second combined it with terms for nanostructure, microheterogeneity, hydrogen-bond networks, supramolecular organization, hydration and water content, viscosity, polarity, and molecular dynamics.

Records were first screened by title and abstract; those retained at this stage were then read in full and included or excluded according to the criteria set out below. The final set of records was analyzed and the evidence synthesized.

Records were included when a compositionally defined eutectic system (stated hydrogen-bond acceptor and donor, with molar ratio) was the entity under evaluation, and when at least one biological, structural or physicochemical property of that system was directly evaluated. Records were not retained when the eutectic system acted only as an extraction or synthesis medium and was removed before evaluation; when the reported effect was attributable solely to a loaded active compound, rather than to the system itself; when the system was not compositionally characterized; or when the subject fell outside the biological field, as in bioremediation or ecological impact assessment.

Search strings and eligibility criteria are provided in Supplemental File S1.

## 2. Reported Biological Effects of DESs

### 2.1. Antimicrobial and Antibiofilm Activity

This section focuses exclusively on the intrinsic antimicrobial and antibiofilm activity of neat DESs, highlighting systems in which eutectic formation promotes biological effects that exceed those of the individual constituents.

Among the biological properties attributed to DESs, antimicrobial activity is arguably the most extensively documented. A wide range of DES formulations, particularly those based on choline chloride, organic acids, terpenes, fatty acids, and quaternary ammonium compounds, have demonstrated inhibitory activity against Gram-positive and Gram-negative bacteria, fungi, and multidrug-resistant microorganisms. Notably, the magnitude and selectivity of these responses frequently diverge from those expected from the isolated constituents, suggesting that biological activity may arise, at least in part, from cooperative interactions established within the eutectic system itself, rather than from simple additive effect [13,14,15].

A recurring observation across in vitro studies in biofilms and planktonic models is the capacity of DESs to perturb microbial envelopes and alter the physicochemical integrity of cellular interfaces. Fatty acid-based DESs, for example, have been shown to exert selective activity against Gram-positive bacteria (*Staphylococcus epidermidis*, *Staphylococcus aureus* Methicillin-resistant strain), while displaying limited efficacy against Gram-negative organisms (*Escherichia coli* and *Pseudomonas aeruginosa*) [16]. This selectivity has generally been attributed to differences in cell envelope architecture, particularly the presence of the lipopolysaccharide-rich outer membrane in Gram-negative species and the distinct lipid composition of bacterial cell walls. However, such explanations remain largely phenomenological and do not fully account for the fact that eutectic formation frequently modifies biological activity relative to the corresponding isolated compounds. Another example is the antifungal effect reported by Kukrić et al. They extensively evaluated the antifungal activity of several hydrophobic DESs against *Monilinia fructicola* and *Botrytis cinerea*, demonstrating significant antifungal activity. However, the observed results were not correlated with the components or the DES structure [17].

Indeed, many antimicrobial DESs were initially developed to exploit advantageous physicochemical characteristics, including low volatility, improved handling, enhanced solubilization capacity, and favorable formulation properties. Unexpectedly, subsequent biological investigations revealed antimicrobial performances that could not always be readily predicted from the known activities of the constituent molecules. This notion is supported by studies reporting biological responses that exceed simple component additivity. For instance, choline geranate, one of the most extensively investigated bioactive DESs, exhibits antimicrobial and antibiofilm in vitro activities against 24- and 72-h-old biofilms of 11 clinically isolated ESKAPE pathogens (defined as *Enterococcus faecium*, *Staphylococcus aureus*, *Klebsiella pneumoniae*, *Acinetobacter baumannii*, *Pseudomonas aeruginosa*, and *Enterobacter* sp.), including multidrug resistant (MDR) isolates. However, the authors could not explain the effects by the known properties of choline or geranic acid alone [18]. These observations challenge a purely compositional interpretation and raise the possibility that eutectic organization itself contributes to microbial inhibition. The same conceptual challenge emerges in antibiofilm research. Several in vitro studies have reported that DESs can disrupt established biofilms, weaken extracellular polymeric substances (EPS), and enhance the activity of conventional antimicrobial agents against tolerant microbial populations [16,19,20].

Mechanistic hypotheses proposed to explain DES-mediated antimicrobial activity include membrane destabilization, osmotic stress induction, modulation of protein conformation, perturbation of hydration equilibria, and disruption of extracellular polymeric matrices. Importantly, many of these mechanisms are inherently linked to the unique physicochemical environment generated by DESs, suggesting that biological activity may emerge not solely from chemical composition, but also from the collective organization of HB, local polarity domains, and water activity gradients [21,22,23]. Such effects have stimulated interest in DESs as potential adjuvants capable of overcoming one of the major limitations of current antibacterial therapies: the persistence of biofilm-associated microorganisms.

Despite the growing body of evidence supporting antimicrobial and antibiofilm efficacy, the field remains largely descriptive. Most mechanistic interpretations continue to rely on the known properties of individual constituents, while direct experimental links between antimicrobial activity and DES micro- or nanostructural organization remain scarce. Consequently, antimicrobial activity exemplifies a broader challenge within DES research: biological effects are consistently observed, yet the structural determinants responsible for these responses remain poorly defined. Establishing such connections represents a critical prerequisite for the development of predictive structure–activity relationships and the rational design of next-generation bioactive eutectic systems.

### 2.2. Cytoprotective and Regenerative Effects

Reports of cytoprotective and regenerative activity span a broad spectrum, ranging from effects plausibly rooted in the eutectic structure to effects that simply reflect an intrinsically bioactive constituent. At one end, the supramolecular organization is itself the active principle: a betaine–urea DES (BU) was designed so that betaine moderates the chaotropic action of urea, yielding a material that selectively de-structures wound slough and biofilm while sparing viable tissue, with biocompatibility confirmed in keratinocytes, preserved fibroblast proliferation and vascularity in vivo, and reduced bacterial load and wound area in a leg ulcer pilot study [24]. Here, a debriding, tissue-sparing function emerges from a minimal two-component eutectic, rather than an added drug, the clearest illustration in this set that a simple, structure-driven system can act directly through its supramolecular organization. Partial structural evidence also appears in menthol-saturated fatty acid DESs: forming the eutectic lowered the cytotoxicity otherwise associated with isolated menthol, and menthol:stearic acid promoted HaCaT keratinocyte migration in a scratch assay, leading the authors to ascribe the improved safety to the supramolecular network [25]. Even so, the regenerative readout is confined to in vitro migration and remains menthol-linked, so the eutectic’s independent contribution is suggested, rather than established. At the other end, the structural contribution is essentially unaddressed because the constituents are, themselves, potent actives. A “bioactive DES” of rosmarinic acid–proanthocyanidin–glycol, sprayed onto monkeypox lesions, scavenged ROS and supported healing while limiting scarring and pigmentation [26], yet rosmarinic acid and proanthocyanidins are established antioxidant and pro-healing phenolics, so the cytoprotection most likely reflects their known pharmacology, rather than eutectic organization. Similarly a choline chloride–ethylene glycol DES ionic gel served as a rapid-gelling hemostatic platform that was also cytocompatible, supporting the viability and proliferation of fibroblasts and keratinocytes [27]. Although the study targets hemostasis, rather than regeneration, the eutectic is not a passive ingredient, but a functional phase of the material, underpinning gelation, adhesion and antibacterial activity, providing a permissive, cytoprotective scaffold in which skin-relevant cells can survive and expand.

Across these studies, regenerative activity is more often implied than demonstrated: most readouts show that cells tolerate and grow on the material, rather than that the eutectic actively drives tissue repair, and where healing-type effects are clearest they coincide with intrinsically bioactive constituents [26]. The open question is therefore not whether DES-based materials are compatible with regenerating tissue, but whether the eutectic structure contributes to repair beyond providing a permissive, cytocompatible environment.

### 2.3. Cryoprotective Effects

A further emerging property is the use of DESs as low-toxicity cryoprotectants positioned as alternatives to dimethyl sulfoxide (DMSO). DESs built from natural metabolites acted as effective vitrification media for mammalian cell lines, matching or exceeding DMSO in L929 cells and, notably, remaining compatible with the growth medium after thawing avoiding the toxic wash-out step DMSO required [27,28]. DES-based formulations preserved human mesenchymal stem cells with recovery equal to or greater than the DMSO gold standard [29]; optimized DESs enabled efficient, low-toxicity preservation of umbilical-cord MSCs [30]; and DESs applied to human sperm preserved viability and chromatin integrity [31]. A structure-relevant signature appears in the altered melting and crystallization behavior of these systems relative to DMSO and water [28], suggesting that the eutectic system modifies ice formation collectively, rather than merely delivering known cryoprotective constituents such as glycerol, proline and sugars, though whether this structural contribution is separable from the constituents remains untested. For deeper reading on the cryoprotectant effect, the reader is referred to Lomba et al. and Marcantonini et al. [32,33].

### 2.4. Cytotoxicity Profiles

Among all biological activities reported for DESs, cytotoxicity provides the most robust experimental framework for investigating whether these systems behave as genuine functional entities, rather than simple mixtures. Unlike studies focused on antimicrobial or regenerative effects, cytotoxicity assessments frequently incorporate constituent controls, concentration–response analyses, and, in some cases, quantitative predictive models. Consequently, this field offers the strongest foundation for evaluating structure–activity relationships in DESs.

A longstanding assumption has been that DESs dissociate completely upon dilution in aqueous environments and that their biological effects therefore reflect only the intrinsic toxicity of their individual constituents. However, an increasing number of studies in both bacterial [14,34,35,36] and eukaryotic cell models [12,37,38,39] challenge this reductionist view by demonstrating that the cytotoxic profiles of DESs often differ significantly from those expected from their starting compounds. As was reported, DES cytotoxicity is highly context-dependent, varying according to HBD and HBA identity, molar ratio, water content, concentration, viscosity, and exposure conditions. Such multidimensional behavior further supports the notion that biological responses arise from the collective physicochemical properties of the eutectic system, rather than from a simple summation of its constituents. These observations suggest that eutectic formation may alter biological behavior in ways that cannot be explained solely by component identity.

Among compositional variables, the HBD appears to exert the strongest influence on cytotoxicity. Hayyan et al. studied organic acid-based DESs and found that acids consistently display the highest toxicity, with malonic acid producing the most cytotoxic choline chloride DES [34], while citric acid-based systems exhibited median effective concentration (EC_50_) values as low as 3.91 mg/mL in HT-29 cells, which were particularly susceptible to acidic formulations [22,40]. Nevertheless, the HBA may become the dominant determinant in systems incorporating bulky quaternary ammonium salts, such as tetrabutylammonium chloride, illustrating that toxicity cannot be attributed to a single structural variable, but instead reflects complex interactions between DES constituents [37].

Attempts to establish predictive cytotoxicity models remain relatively scarce and continue to rely primarily on constituent-based molecular descriptors. To our knowledge, the only Quantitative Structure–Activity Relationship (QSAR) model specifically developed for DES cytotoxicity was reported by Ahmadi et al. [41]. Using choline chloride-based DESs, Ahmadi et al. predicted IC_50_ values from constitutional, topological, molecular walk count, and physicochemical descriptors of the individual components plus the HBA:HBD molar ratio, yielding a model able to estimate the cytotoxicity of choline chloride-based DESs. Although the resulting model successfully estimated cytotoxicity (IC_50_ = 3.52–75.46 mM in HEK-293 cells), it did not incorporate descriptors of the eutectic assembly itself, such as HB organization, nanostructure, or collective physicochemical properties. Consequently, the current generation of QSAR models remains fundamentally composition-based, rather than structure-based.

Interpretation of cytotoxicity data across studies should nevertheless be approached cautiously. Differences in cell models (HEK-293, HaCaT, HT-29, Caco-2, HeLa, 3T3-L1), viability assays, water content, exposure conditions, and DES preparation protocols limit direct comparison of toxicity rankings between independent investigations. As a result, cytotoxicity trends are generally reliable within individual studies, but remain difficult to extrapolate across the broader literature.

Of particular relevance is the recurring finding that eutectic formation attenuates toxicity relative to the isolated constituents, rather than the toxicities combining additively. An example is the work reported by Dubinenko G et al., who found that DES showed EC50 values higher than aqueous solutions of their individual components, and explained the observation by a partial stabilization of the acidic components and reduced proton release [42]. Comparable findings have been reported for betaine-based DESs developed as enzyme-stabilizing media, which exhibited lower cytotoxicity than their isolated constituents, while preserving the catalytic activity and thermal stability of α-glucosidase [43].

Collectively, biological effect observations (Table 1) challenge the assumption that DES cytotoxicity can be predicted solely from the properties of their constituent molecules. Instead, they suggest that eutectic formation modifies the physicochemical environment experienced by biological systems, leading to non-additive cellular responses.

Despite efforts to systematically investigate the influences of some physicochemical properties such as viscosity, density, surface tension and pH on activity, the authors recognize limitations due to a lack of understanding of how their molecular compositions determine biological activity [42]. In this context, current predictive frameworks continue to ignore descriptors related to supramolecular organization, HB dynamics, nanostructural heterogeneity, and hydration-dependent restructuring. Incorporating these hierarchical structural features into future quantitative models represents a critical step toward the development of truly predictive structure–activity relationships for bioactive DESs.

The critical question emerging from the previous sections is therefore not whether DESs can exert biological effects, but whether these effects can be linked to specific structural features of the eutectic system itself. Addressing this question requires moving beyond composition and examining the multiscale organization of DESs.

## 3. Fundamental Micro- and Nanostructural Aspects of DESs

### 3.1. DESs as Dynamic Networks

Understanding the biological activity of DESs requires first recognizing that these systems are not static molecular mixtures, but dynamically organized physicochemical networks. Their structural organization is governed by an intricate balance of hydrogen bonding, electrostatic interactions, and van der Waals forces that collectively generate physicochemical properties distinct from those of the isolated constituent. While the pronounced depression of the melting point remains the defining characteristic of DES formation, increasing evidence indicates that the same intermolecular interactions responsible for eutectic behavior also establish a dynamic structural framework that may ultimately govern biological function [44,45,46]. The molecular organization of DESs has been extensively investigated using complementary computational and experimental approaches. Molecular dynamics simulations and quantum chemical calculations have revealed highly cooperative HB networks, extensive charge delocalization, and continuous molecular rearrangements, particularly in choline chloride-based systems [47,48,49]. These computational observations are supported by experimental evidence obtained through neutron total scattering and quasi-elastic neutron scattering (QENS). In this context, Hammond OS et al. [47] revealed that the DES structure is mostly retained upon hydration, with water acting both as a secondary smaller HBD, and forming transient wormlike aggregates. Rather than behaving as homogeneous liquids, DESs frequently exhibit nanoscale heterogeneity characterized by dynamic domains enriched in distinct molecular species [48,49]. These findings challenge the long-held assumption that DESs behave as homogeneous liquids and instead support a model in which they comprise dynamically evolving molecular environments.

One of the most compelling demonstrations of this structural plasticity was provided by Araujo et al., who showed that urea undergoes a conformational transition upon eutectic formation with choline chloride. Whereas crystalline urea adopts a predominantly planar (sp^2^-like) geometry, incorporation into the DES induces a non-planar (sp^3^-like) conformation, reflecting the extensive reorganization of intermolecular interactions required to stabilize the HB network [50]. This observation illustrates that eutectic formation involves more than molecular association; it actively reshapes the conformational landscape of the constituent molecules, generating a flexible and adaptive network capable of continuous structural rearrangement.

Importantly, these dynamic structural features give rise to emergent physicochemical properties that cannot be predicted from the isolated components alone. Glyceline, the eutectic mixture formed by choline chloride and glycerol (1:2 molar ratio), provides a representative example. Despite containing glycerol as its major constituent, glyceline exhibits a substantially lower viscosity than pure glycerol, a behavior attributed to enhanced long-range diffusivity within the reorganized HB network [51]. Such observations illustrate that collective molecular organization can profoundly modify bulk physicochemical behavior, reinforcing the concept that DES properties emerge from network architecture, rather than simple compositional averaging.

Collectively, these findings redefine DESs as adaptive molecular networks characterized by continuous structural fluctuations across multiple length scales. Rather than representing homogeneous solvent phases, DESs comprise dynamic assemblies whose local organization evolves in response to environmental conditions such as hydration, temperature, and molecular composition. This structural organization provides the physicochemical basis for many of the emergent biological effects discussed throughout this review and establishes the conceptual foundation for moving beyond composition-based interpretations toward structure-dependent models of DES bioactivity.

### 3.2. Water-Mediated Structural Rearrangement

Water is now recognized as an active structural component of DESs, rather than a simple diluent. Instead of merely reducing viscosity or weakening intermolecular interactions, water dynamically reorganizes the HB network, driving transitions between distinct structural states while modulating the physicochemical properties experienced by dissolved molecules [47,48]. Consequently, hydration should be regarded as a process of structural rearrangement, rather than one of complete structural disruption. Experimental and computational studies indicate that the effect of water is highly dependent on its concentration. At low hydration levels, water molecules become incorporated into the existing HB network, acting as secondary HBD and HBA while preserving much of the local molecular organization. As hydration increases, progressive reorganization of intermolecular interactions occurs, accompanied by changes in viscosity, diffusivity, and molecular mobility [52]. Importantly, these transitions do not necessarily result in complete disassembly of the eutectic structure. Instead, increasing evidence suggests that hydrated DESs retain localized molecular organization in the form of transient clusters, heterogeneous nanodomains, and dynamically reorganized HB networks, particularly in systems containing choline chloride and amphiphilic constituents [53]. Experimental observations support this interpretation. Nuclear magnetic resonance (NMR) and rotating-frame Overhauser effect spectroscopy (ROESY) analyses have demonstrated that labile proton correlations characteristic of the eutectic network persist at physiologically relevant water contents (approximately 10–30% *w*/*w*), indicating that key intermolecular interactions remain preserved despite substantial dilution [54].

Similar findings have been reported for amphiphile-containing DESs, where hydration promotes structural reorganization, rather than complete dissociation, of the molecular network [55]. Collectively, these studies suggest that hydration generates new dynamic equilibrium states, in which structural organization is modified but not entirely lost.

This distinction has important implications for understanding DES bioactivity. Most biological experiments are performed under highly hydrated conditions, whereas most physicochemical characterization studies continue to investigate neat or minimally hydrated DESs. Consequently, the structural descriptors currently used to interpret biological responses often correspond to physicochemical states that differ substantially from those encountered in biological environments. This discrepancy represents a major conceptual gap in the field and likely contributes to the limited mechanistic understanding of DES-mediated biological activity. Rather than asking whether DESs retain their original structure after dilution, future studies should seek to identify which structural features persist, how hydration reshapes local molecular organization, and which of these hydration-dependent architectures are biologically relevant. Resolving these questions will be essential for establishing meaningful structure–activity relationships under physiological conditions and for advancing the rational design of bioactive eutectic systems.

Molecular modeling is expected to play a central role in elucidating how hydration reshapes the supramolecular organization of DESs under physiologically relevant conditions [56]. Molecular dynamics simulations and complementary multiscale computational approaches can reveal hydration-dependent hydrogen-bond rearrangements, nanodomain formation, molecular mobility, and interactions with biomolecules at spatial and temporal resolutions that remain challenging to capture experimentally [57]. When complemented by quantum mechanical calculations, these approaches provide atomistic insight into descriptors such as hydrogen-bond lifetimes, coordination patterns, solvent-accessible surface area (SASA), conformational flexibility, interaction energies, and local hydration [58]. Together, these parameters offer a mechanistic link between nanoscale solvent organization and biological responses. Thus, molecular modeling is emerging as a powerful tool for identifying predictive supramolecular descriptors that can support the rational design of DES platforms operating under biological conditions.

## 4. DES Biological Effects Through Cellular Microenvironment Modulation: The Missing Link Between Structure and Activity

The biological activity of DESs may not arise exclusively from their molecular composition, but also from the unique physicochemical microenvironments generated by their dynamic structural organization. Unlike conventional aqueous solutions, DESs constitute organized solvent systems in which HB networks, molecular clustering, and hydration dynamics collectively shape the local environment experienced by biomolecules. From this perspective, DESs should be viewed not simply as solvent media, but as adaptive physicochemical environments capable of modulating biological interactions.

A growing body of physicochemical evidence supports this interpretation. Multiple independent studies have demonstrated the persistence of structural features following hydration, including the retention of anomalous viscosity behavior after dilution [59], preservation of nanostructural organization with increasing water content [47], persistence of spectroscopic signatures associated with intermolecular interactions [55], and hydration-induced structural reorganization, rather than complete disruption, of the HB network [60]. Collectively, these observations indicate that dilution does not simply eliminate eutectic organization, but instead generates new hydration-dependent structural states that remain distinct from conventional aqueous solutions.

These dynamic architectures create heterogeneous physicochemical microenvironments in which biomolecules experience local conditions that differ substantially from the bulk properties of the system. Rather than sensing average viscosity or polarity, dissolved molecules interact with domain-specific environments characterized by localized viscosity, polarity fluctuations, hydrogen-bond density, and microheterogeneity [59,60,61,62,63,64,65]. Such localized environments are continuously reshaped through the formation and disruption of hydrogen-bond networks, suggesting that biological responses may be governed by the physicochemical conditions experienced at the molecular scale, rather than by the average properties of the DES as a whole.

Water activity emerges as a particularly important physicochemical variable linking DES structure to biological function. Extensive HB interactions reduce the availability of free water while simultaneously reorganizing hydration dynamics. These changes influence osmotic pressure, protein hydration shells, conformational flexibility, and intermolecular recognition, all of which are fundamental determinants of biomolecular stability and cellular function [66]. Consequently, DESs may regulate biological systems not only through direct molecular interactions, but also by altering the physicochemical landscape in which those interactions occur.

Additional biophysical mechanisms may further contribute to this phenomenon. Variations in localized polarity, osmotic gradients, molecular crowding, and the chaotropic–kosmotropic balance are all expected to influence protein folding, membrane organization, enzyme activity, and macromolecular assembly [67,68]. Although these mechanisms remain largely unexplored in DES research, they provide plausible explanations for many of the emergent biological effects discussed throughout this review and represent promising directions for future mechanistic investigation.

Distinguishing solvent-mediated effects from structural effects nevertheless remains experimentally challenging. Hydration, temperature, and compositional changes simultaneously modify HB organization and bulk solvent properties, making it difficult to isolate the contribution of each factor independently [69]. This limitation highlights one of the major conceptual gaps in current DES research. Rather than considering solvent properties and structural organization as independent variables, future studies should investigate how dynamic structural rearrangements generate localized physicochemical microenvironments that ultimately govern biological functions. Such an approach may provide the missing mechanistic link between DES structure and biological activity and establish the foundation for predictive Hierarchical Structure–Activity Relationships (H-SAR) in bioactive eutectic systems.

## 5. DESs as Emergent Functional Systems

The central question emerging from current DES research is whether these systems should be regarded simply as mixtures of known compounds, or as emergent functional entities possessing physicochemical and biological properties that cannot be predicted from their isolated constituents. This distinction extends beyond semantics and fundamentally influences how DESs should be investigated, interpreted, and ultimately translated into biomedical applications.

The concept of DESs as new functional entities was proposed during the early development of the field [70] and has gained increasing support from a growing body of physicochemical evidence. Unlike conventional mixtures, DESs exhibit different and new properties arising from cooperative intermolecular interactions, including pronounced melting point depression, non-ideal thermodynamic behavior, persistent hydrogen-bond organization, hydration-dependent structural rearrangement, and adaptive molecular networks. Together, these characteristics suggest that eutectic formation promotes a functional identity that extends beyond the intrinsic properties of the individual constituents. The biological evidence reviewed throughout this article further reinforces this perspective. DESs frequently display non-additive antimicrobial, cytotoxic, and regenerative activities that cannot always be rationalized by the known pharmacology or biological behavior of their isolated components. Rather than acting solely through the chemical identity of their constituents, these systems appear capable of generating localized physicochemical microenvironments that modulate biomolecular interactions, membrane behavior, protein hydration, and cellular responses. Such observations are consistent with the hypothesis that biological activity is related with the collective organization of the eutectic system, rather than from composition alone.

This conceptual framework has important implications for pharmaceutical sciences, biomaterials engineering, toxicology, and pharmacology. The debate has become particularly relevant for THEDES, in which active pharmaceutical ingredients are intentionally incorporated as eutectic constituents. In these systems, eutectic formation may substantially modify pharmacokinetic and pharmacodynamic behavior, raising the question of whether THEDES should be considered solely as combinations of approved active pharmaceutical ingredients and excipients, or as novel functional entities requiring independent characterization [71]. Similar considerations apply to safety assessment, where current toxicological strategies remain undistinguished from the classical component-based ones [72].

Consequently, the absence of standardized methodologies for characterizing DES organization under biologically relevant conditions represents one of the principal barriers to their clinical translation [73]. Current evaluation frameworks remain largely focused on chemical composition, while descriptors capable of capturing supramolecular organization, hydration-dependent restructuring, dynamic microenvironment formation, and emergent physicochemical properties are rarely incorporated into biological assessment. This disconnect limits mechanistic interpretation and hinders the development of predictive models for DES design.

From this perspective, DESs may be considered as new functional chemical entities, whose identity emerges from collective molecular organization, and whose biological properties arise from dynamic physicochemical interactions operating across multiple structural scales. Recognizing this hierarchical organization provides the conceptual basis for moving beyond empirical formulation toward predictive Hierarchical Structure–Activity Relationships (H-SAR), thereby enabling the rational engineering of next-generation bioactive eutectic systems.

## 6. Toward Predictive Structure–Activity Relationships in DESs

**Figure 2:**
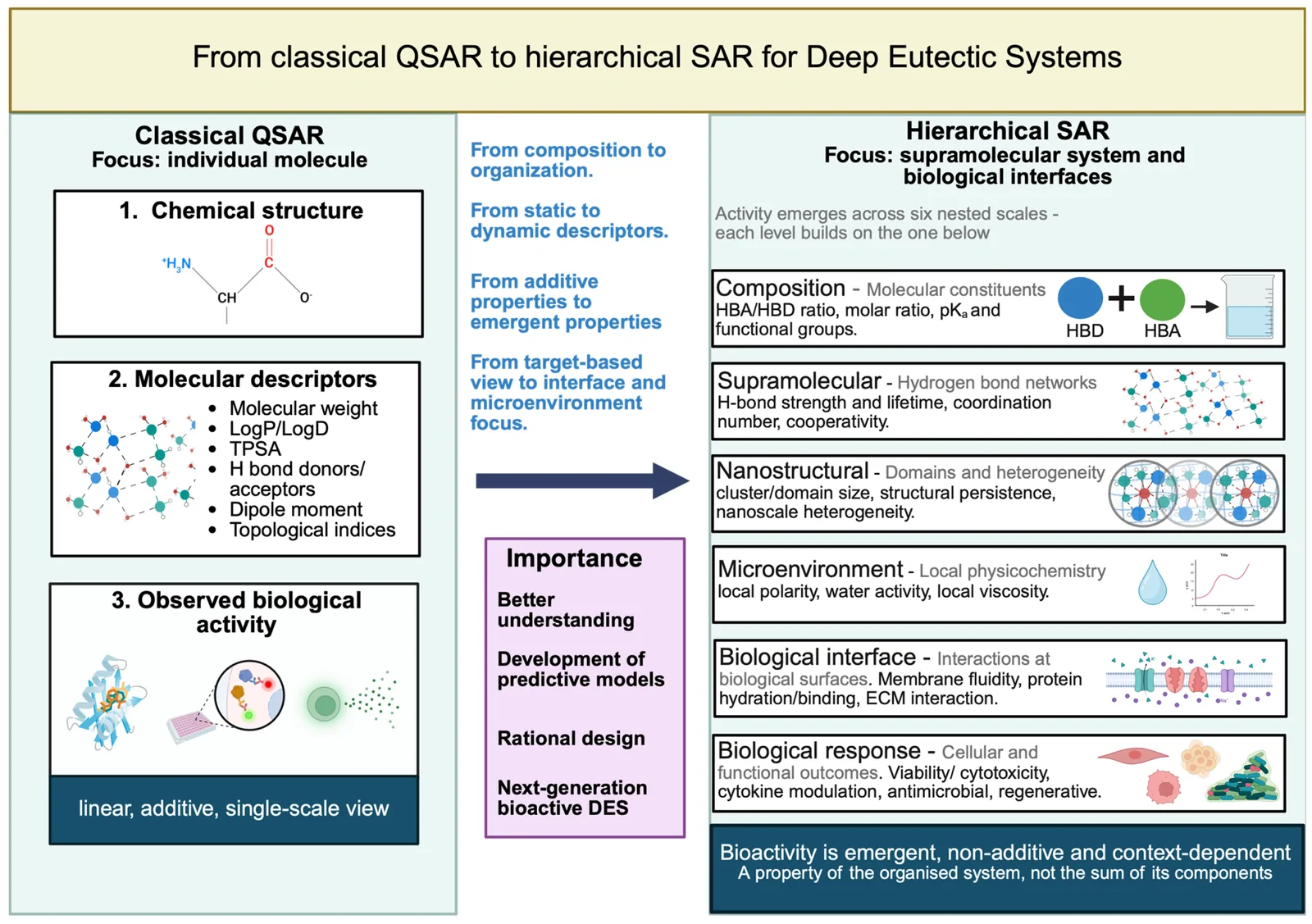
Composition-based Structure–Activity Relationships toward Hierarchical Structure–Activity Relationships (H-SAR) for Deep Eutectic Systems. Created in BioRender. UDD, D. (2026) https://BioRender.com/rxsuosp.

Traditional Structure–Activity Relationship (SAR) and Quantitative Structure–Activity Relationship (QSAR) approaches were developed for discrete molecular entities, whose biological activity can be related to well-defined molecular descriptors for constitutional, topological, geometric, electronic, and physicochemical properties [71,72]. These descriptors have been highly successful in medicinal chemistry because biological function is directly associated with individual molecular structures. DESs present additional challenges because their physicochemical properties emerge from dynamic intermolecular interactions, rather than from isolated molecular structures alone. Numerous studies reviewed here show that eutectic formation frequently alters physicochemical and biological properties relative to those of the individual constituents, suggesting that conventional molecular descriptors may not fully capture the determinants of DES bioactivity. Based on these observations, we propose a Hierarchical Structure–Activity Relationship (H-SAR) as a conceptual framework to organize the different levels of structural information that may contribute to DES biological activity. Rather than replacing conventional molecular descriptors, H-SAR extends them by incorporating descriptors associated with supramolecular organization, including hydrogen-bond networks, nanostructural heterogeneity, hydration-dependent structural persistence, and localized physicochemical microenvironments (Figure 2). These descriptors may ultimately provide a mechanistic link between supramolecular organization and biological function by influencing molecular and cellular processes involved in extracellular matrix organization, regenerative microenvironments, and tissue-level biological responses [52,74,75,76].

Several related research fields, including supramolecular chemistry, self-assembled biomaterials, colloidal systems, and ionic liquids, have progressively incorporated descriptors beyond molecular composition to explain emergent functional behavior [73,74]. These developments provide useful conceptual precedents for exploring similar multiscale approaches in DES research.

At present, H-SAR should be regarded as a qualitative conceptual framework, rather than a predictive methodology. Future progress will depend on the availability of standardized experimental datasets integrating molecular composition, supramolecular descriptors (e.g., hydrogen-bond dynamics, cluster organization, local polarity, diffusion, viscosity, and water activity), and biological endpoints.

Although substantial methodological challenges remain—including the experimental characterization of DES supramolecular organization and the development of computational models capable of describing highly non-ideal systems—the framework proposed here provides a possible perspective for integrating physicochemical organization with biological function.

Importantly, the H-SAR framework proposed here should not be interpreted as direct evidence that supramolecular organization is the sole mechanistic origin of DES biological activity. Rather, it provides a conceptual model that integrates multiple interconnected levels of organization—from molecular composition and supramolecular architecture to emergent physicochemical properties and biological responses. Within this framework, supramolecular organization is proposed as one plausible mechanistic contributor that may directly or indirectly influence biological function by modulating properties such as solubility, ionization, chemical activity, membrane permeability, partitioning, local concentration, and molecular interactions in biologically relevant environments. Together, these variables may provide a more mechanistically meaningful representation of the biological identity of DESs than composition alone [77]. Establishing causal relationships among these hierarchical levels will require future studies combining advanced structural characterization, molecular modeling, and rigorous constituent-matched biological controls. As sufficient standardized datasets of variables become available, these descriptors could be progressively incorporated into multivariate statistical or machine-learning models. Thus, the H-SAR framework proposed in this review could evolve from a qualitative multiscale conceptual framework to a fully quantitative predictive methodology, analogous to the historical evolution of classical QSAR.

## 7. Conclusions and Future Perspectives

DESs have evolved from being regarded as sustainable solvent alternatives to emerging as dynamic supramolecular systems with the capacity to modulate biological function. The growing body of evidence reviewed here demonstrates that DESs influence antimicrobial activity, inflammation, oxidative stress, tissue repair, and cellular behavior through mechanisms that frequently cannot be fully explained by the intrinsic properties of their isolated constituents. Collectively, these observations challenge the traditional composition-based interpretation of DES bioactivity and instead support the view that biological responses arise, at least in part, from emergent physicochemical organization.

Despite these advances, a fundamental gap remains between the extensive characterization of DES physicochemical properties and the mechanistic understanding of their biological activity. Current studies continue to rely predominantly on compositional screening strategies, while the potential contribution of hydrogen-bond organization, hydration-dependent structural rearrangement, nanostructural heterogeneity, localized physicochemical microenvironments, and structural persistence under biologically relevant conditions remains insufficiently explored. Consequently, the structural determinants governing DES-mediated biological effects remain largely undefined.

Bridging this gap will require a shift in both methodology and conceptual framework. Future research should integrate advanced physicochemical characterization, multiscale computational modeling, and biologically relevant experimental systems to identify structural descriptors capable of predicting biological behavior. Rather than treating physicochemical characterization and biological evaluation as independent disciplines, these approaches should be combined into unified experimental frameworks that investigate DES organization under physiological conditions.

The evidence synthesized throughout this review further suggests that DESs are more appropriately viewed as dynamic functional chemical entities, whose biological identity emerges from collective molecular organization, rather than from chemical composition alone. This perspective aligns DES research with broader developments in supramolecular chemistry, systems chemistry, biomaterials science, and nanomedicine, where function increasingly arises from hierarchical organization, rather than individual molecular components.

Based on the current state of knowledge, we propose that future DES research should move beyond conventional composition-based Structure–Activity Relationships toward Hierarchical Structure–Activity Relationships (H-SAR). Within this framework, biological activity is understood as the consequence of successive levels of organization, extending from molecular composition and hydrogen-bond networks to hydration-dependent restructuring, localized physicochemical microenvironments, biomolecular interactions, and, ultimately, biological function. Such a transition provides not only a mechanistic explanation for the emergent biological properties of DESs, but also a roadmap for the rational design of next-generation bioactive eutectic systems with tunable antimicrobial, immunomodulatory, regenerative, and therapeutic functions.

Ultimately, the future of DES research may depend less on discovering new component combinations than on understanding—and intentionally engineering—the dynamic structural organization that influences their biological identity. Recognizing this paradigm shift has the potential to transform DESs from empirically optimized formulations into predictively designed functional systems for biomedical applications.

## Figures and Tables

**Table 1:** Composition, reported bioactivity, and constituent-level comparison across 9 DES/NADES studies.

DES Composition	Molar Ratio/ Water Content	Biological Model	DES Role	Reported Effect	Proposed Mechanism	Constituent Comparison	Ref
**28** **ChCl-based NADESs.** **HBDs: polyols (ethylene glycol, glycerol, butanediol, sorbitol, xylitol) and sugars (glucose, fructose, sucrose, raffinose).**	ChCl:HBD 1:4 to 9:1; (e.g., ChCl:glycerol 1:2–1:4). Water content: 0.00–0.35 wt%, depending on NADES.	In vitro: HEK-293 cells	Cytotoxicity and QSAR modeling linking NADES composition/structure to cell toxicity.	Cytotoxic to HEK-293 (IC_50_ 3.52–75.46 mM, 72 h MTT). More toxic than isolated constituents, far less than imidazolium IL [C8mim]Cl 0.02 mM. Toxicity varied with HBD identity and HBA:HBD ratio.	QSAR: cytotoxicity rose with rotatable bond number and atomic van der Waals volume, and fell with ChCl:HBD ratio and HBD carbon number.	NADES more cytotoxic than components (synergistic toxicity).	Ahmadi et al. 2018 [41]
**Six αHA-based DESs: glycolic/lactic/tartaric acid with choline chloride or tetraethylammonium chloride.**	HBA:HBD 1:2. Water content: GA-DES 17–18, LacA-DES ~9, TA-DES 14 wt%.	In vitro: *S. aureus*, MRSA, *E. coli*, *P. aeruginosa*; *P. aeruginosa* biofilms; 3T3-L1 fibroblasts.	Tailorable antimicrobial/antibiofilm DES, less cytotoxic than free α-hydroxy acids.	Broad-spectrum antibacterial (MIC 4.57–9.08 mg/mL vs. S. *aureus*, MRSA, *E. coli*, *P. aeruginosa*) Low cytotoxicity (3T3-L1 IC_50_ 27.7–37.6 mg/mL), less cytotoxic than free αHAs.	Acidity drives antibacterial/biofilm disruption (SEM-confirmed). Eutectic H-bonding stabilizes the acid, lowering cytotoxicity and decoupling potency from toxicity.	DES attenuates cytotoxicity versus free αHAs (reduced free-acid availability).	Dubinenko et al. 2025 [42]
**Fifteen DESs based on [Chol]Cl, [N1111]Cl, or [N4444]Cl as HBA, combined with butanoic acid, hexanoic acid, ethylene glycol,** **1-propanol, or urea as HBD.**	HBA:HBD 1:1 Water content measured (Karl Fischer), although numeric values were not reported.	In vitro: HaCaT human keratinocytes; MNT-1 human melanoma cells.	Cytotoxicity/ biocompatibility screening of DESs for skin cosmetic/pharmaceutical use.	Most [Chol]Cl- and [N1111]Cl-based DESs were non-cytotoxic up to 500 µg/mL after 72 h. Several [Chol]Cl- and [N1111]Cl-based DESs increased HaCaT viability. All [N4444]Cl-based DESs were cytotoxic in both cell lines.	Toxicity pattern attributed mainly to [N4444]Cl as HBA, since [N4444]Cl-based DESs resembled the toxicity profile of isolated [N4444]Cl.	HBAs, HBDs, DESs tested individually; toxicity tracks [N4444]Cl acceptor (additive).	Macário et al. 2019 [37]
**ChCl:fructose and ChCl:glucose NADESs (2:1); DAC:TEG DES (1:3).**	ChCl:Fru 2:1; ChCl:Glu 2:1; DAC:TEG 1:3 Water content not reported.	In vitro: HelaS3, PC3, A375, AGS, MCF-7, and WRL-68 cells. In vivo: ICR mice for acute toxicity testing.	Cytotoxicity/anticancer screening of binary NADES compared with a non-natural DES.	Cytotoxic to five cancer lines and normal hepatic cells; NADES less toxic than the synthetic DES. Fructose-NADES more toxic than glucose-NADES. In vivo (mice): reversal: NADES more toxic than the DES, inducing liver failure.	NADES increase membrane porosity and redox stress. Sugars form advanced glycation end products (AGEs) driving ROS with fructose more ROS-generating than glucose.	Components and combinations tested at in-DES concentrations; NADES toxicity additive.	Mbous et al. 2017 [12]
**Menthol-based NADESs with saturated fatty acids: Me:LA (4:1; also 2:1/8:1), Me:MA 8:1, Me:SA 8:1.**	Me:LA 4:1; Me:MA 8:1; Me:SA 8:1 Water content not reported. Hydrophobic menthol:fatty acid DES; water is not a design component.	In vitro: *S. aureus*, MRSA, MRSE, *E. coli*, *P. aeruginosa*, *C. albicans*; HaCaT keratinocytes.	Antimicrobial/antibiofilm eutectic formulations with potential use in wound healing.	Antimicrobial against Gram-positives (*S. aureus*, *MRSA*, *MRSE*) and C. albicans, not Gram-negatives. Me:LA 4:1 disperses/removes biofilms (*E. coli*, *MRSA*, *C. albicans*). Non-cytotoxic to keratinocytes.	Membrane dissolution by menthol/fatty acids; eutectic structure boosts constituent bioavailability; Me:LA 4:1 disrupts biofilm.	Constituents tested alone versus DES; antimicrobial tracks menthol, cytotoxicity is synergistic.	Oliveira et al. 2023 [16]
**Betaine:urea NADES, named BU.**	Betaine:urea 1:1.5 14% *w/w* water (Karl Fischer).	In vitro artificial slough (debridement, mixed biofilm). Ex vivo human skin. In vivo murine diabetic wounds, Pilot clinical trial.	Non-biocidal topical NADES for dual debridement and antibiofilm activity in chronic wound treatment.	Effective artificial slough disorganization: matrix organization reduced to 56 ± 3.5% after 15 min. Clinical slough: BU induced marked swelling and complete sample disintegration before 120 min. Biocompatible, non-pro-inflammatory.	Non-biocidal: betaine-modulated chaotropic/keratolytic urea plus high hygroscopicity draws water, disorganizing the proteinaceous slough matrix. Antibiofilm via physical dispersal; requires the eutectic system.	Debridement attenuated vs. urea (betaine-modulated); antibiofilm emergent, absent when separated.	Schuh et al. 2024 [24]
**Ten NADESs from choline chloride, betaine, or citric acid (HBAs) with organic acids, sugars, sugar alcohols, proline, glycerol or urea (HBDs)**	Variable (1:1 to 5:2; ternary systems 1:1:1). Betaine:Malic acid:Proline (BMaPro) among them at a 1:1:1 molar ratio. Tested at 10, 30 and 50% *w/w* water.	In vitro: *E. coli*, *P. mirabilis*, *S. typhimurium*, *P. aeruginosa*, *S. aureus*, *C. albicans*; HeLa, MCF-7, HEK293T cells	Biological profiling of NADESs for antimicrobial, cytotoxic, and antioxidant activity.	Antimicrobial and cytotoxic only for organic acid NADES (oxalic strongest). Sugar/polyol/urea systems benign. Some antioxidant (malic-rich) and pro-proliferative (BMaPro) effects.	Acidic HBDs drive toxicity/antimicrobial activity (low pH, membrane damage). NADESs more active than isolated acids (synergism).	Components tested alone; acid-NADES more antimicrobial (synergism); malic toxicity masked within BMaPro.	Radošević et al. 2018 [22]
**Acetylcholine chloride: acetamide (HBA:HBD), 1:2.**	AcChCl:acetamide 1:2. Water content not reported.	In vitro: bacterial system *E. coli BL21(DE3).*	Model DES to evaluate toxicity testing methodology and microbial tolerance.	Non-toxic to *E. coli* ≤ 300 mM; tolerated 300–450 mM (preadapted cells). Toxic ≥ 600 mM (>30% inhibition, diauxic growth); complete inhibition at 750 mM.	Toxicity above 600 mM driven not only by DES composition, but by media acidification from DES hydrolysis during growth.	Both constituents tested individually at their in-DES concentrations. Eutectic more toxic than either component alone—synergistic toxicity.	Torregrosa-Crespo et al. 2020 [36]
**Nine betaine-based NADESs: betaine (HBA) with ethylene glycol or glycerol (HBD), at molar ratios 1:3–1:6 (Bet:EG) and 1:2–1:6 (Bet:Gly).**	Bet:EG 1:3, 1:4, 1:5, 1:6. Bet:Gly 1:2, 1:3, 1:4, 1:5, 1:6. Water content not reported.	In vitro: HaCaT and HeLa, plus a cell-free α-glucosidase enzyme activity/stability assay.	Biocompatible DES media probing cytotoxicity, inflammatory gene expression, and α-glucosidase activity/stability.	Low cytotoxicity in HeLa/HaCaT. Concentration-dependent pro-/anti-inflammatory gene modulation. Every DES enhanced α-glucosidase activity (>120%) and thermal storage stability.	Betaine–polyol hydrogen-bond network reshapes the enzyme’s microenvironment, stabilizing its active conformation.	DES less cytotoxic than pure betaine. The polyol HBD dilutes betaine’s membrane interactions, lowering cytotoxicity relative to pure betaine.	Zuriaga et al. 2025 [43]

Abbreviations. General (used throughout the table): DES, deep eutectic system; NADES, natural deep eutectic system; HBA, hydrogen-bond acceptor; HBD, hydrogen-bond donor. DES Compositions: ChCl (also written [Chol]Cl), choline chloride; [N1111]Cl, tetramethylammonium chloride; [N4444]Cl, tetrabutylammonium chloride; αHA, α-hydroxy acid; GA, glycolic acid; LacA, lactic acid; TA, tartaric acid; Me, menthol; LA, lauric acid; MA, myristic acid; SA, stearic acid; BU, betaine:urea NADES; DAC, N,N-diethylethanolammonium chloride; TEG, triethylene glycol; EG, ethylene glycol; Bet, betaine; Gly, glycerol; BMaPro, betaine:malic acid:proline (ternary NADES); AcChCl, acetylcholine chloride. Molar Ratio/Water Contents: Fru, fructose; Glu, glucose; wt%, weight percent; *w*/*w*, weight per weight; Karl Fischer, Karl Fischer titration (water content method). Biological Model: HEK-293/HEK293T, human embryonic kidney cell lines; HaCaT, immortalized human keratinocyte line; MNT-1, human melanoma line; HeLa/HeLaS3, human cervical carcinoma line; PC3, human prostate carcinoma line; A375, human melanoma line; AGS, human gastric adenocarcinoma line; MCF-7, human breast adenocarcinoma line; WRL-68, human hepatic line; 3T3-L1, murine embryonic fibroblast line; ICR, Imprinting Control Region, (outbred) mice; *E. coli* BL21(DE3), *Escherichia coli* protein-expression strain; MRSA, methicillin-resistant Staphylococcus aureus; MRSE, methicillin-resistant *Staphylococcus epidermidis*. Microbial species: *S. aureus* (*Staphylococcus aureus*), *S. epidermidis* (*Staphylococcus epidermidis*), *E. coli* (*Escherichia coli*), *P. aeruginosa* (*Pseudomonas aeruginosa*), *P. mirabilis* (*Proteus mirabilis*), *S. typhimurium* (*Salmonella typhimurium*), *C. albicans* (*Candida albicans*). Reported Effects: IC50, half-maximal inhibitory concentration; MIC, minimum inhibitory concentration; MTT, 3-(4,5-dimethylthiazol-2-yl)-2,5-diphenyltetrazolium bromide viability assay; IL, ionic liquid; [C8mim]Cl, 1-octyl-3-methylimidazolium chloride; mM, millimolar. Proposed Mechanism: QSAR, Quantitative Structure–Activity Relationship; ROS, reactive oxygen species; SEM, scanning electron microscopy; AGEs, advanced glycation end products.

## Data Availability

The original contributions presented in this study are included in the article/Supplementary Material. Further inquiries can be directed to the corresponding author.

## Supplementary Materials

The following supporting information can be downloaded at: https://www.mdpi.com/article/10.3390/pharmaceutics18080990/s1, File S1. Search strategy and eligibility criteria.

## Abbreviations

AcChCl, Acetylcholine chloride; AGE, Advanced glycation end product; AMPS 2-, Acrylamido-2-methylpropanesulfonic acid; API, Active pharmaceutical ingredient; AscA, Ascorbic acid, Bet, Betaine; BMaPro, Betaine–malic acid–proline natural deep eutectic system; BU, Betaine–urea natural deep eutectic system; CCL, Choline chloride–lactic acid natural deep eutectic system; CCM, Choline chloride–malic acid natural deep eutectic system; ChCl, Choline chloride; [Chol]Cl, Choline chloride; CitA, Citric acid; MC-Na, Sodium carboxymethylcellulose; COX-2, Cyclooxygenase-2; COSMO-RS, Conductor-like screening model for real solvents; DAC, N,N-Diethylethanolammonium chloride; DADMAC, Diallyldimethylammonium chloride; DES, Deep eutectic system; DMSO, Dimethyl sulfoxide; DSC, Differential scanning calorimetry; EC_50_, Half-maximal effective concentration; EG, Ethylene glycol; EPS, Extracellular polymeric substances; FTIR, Fourier-transform infrared spectroscopy; Fru Fructose; FXII, Coagulation factor XII; GA, Glycolic acid; Glu, Glucose; Gly, Glycerol; HB, Hydrogen bond; HBA, Hydrogen-bond acceptor; HBD, Hydrogen-bond donor; HCE, Human corneal epithelial cell; HEC, Hydroxyethyl cellulose; H-SAR, Hierarchical structure–activity relationship; HUVECs, Human umbilical vein endothelial cells; IC_50_, Half-maximal inhibitory concentration; IL, Ionic liquid; LA, Lauric acid; LacA, Lactic acid; Lin, Linalool; LOAEL, Lowest-observed-adverse-effect level; MA, Myristic acid; MalA, Malic acid; MaloA, Malonic acid; MDR-TB, Multidrug-resistant tuberculosis; Me, Menthol; MIC, Minimum inhibitory concentration; MM, Matrix metalloproteinase; MRSA, Methicillin-resistant Staphylococcus aureus; MTT, 3-(4,5-Dimethylthiazol-2-yl)-2,5-diphenyltetrazolium bromide; NADES, Natural deep eutectic system; NMR, Nuclear magnetic resonance; NOAEL, No-observed-adverse-effect level; NSAID, Non-steroidal anti-inflammatory drug; PBS, Phosphate-buffered saline; POM, Polarized optical microscopy; QENS, Quasi-elastic neutron scattering; QSAR, Quantitative structure–activity relationship; RBC, Red blood cell; Reline_FD, Freeze-dried reline composed of choline chloride and urea; Reline_H, Heat-prepared reline composed of choline chloride and urea; RMM, Residual molecular mobility; ROESY, Rotating-frame Overhauser effect spectroscopy; ROS, Reactive oxygen species; RP, Recrystallization propensity; SA, Stearic acid; Saf, Safranal SAR, Structure–activity relationship SDD, Spatial domain distribution; SRB, Sulforhodamine B; TA, Tartaric acid; TEG, Triethylene glycol; Tg, Glass transition temperature; THEDES, Therapeutic deep eutectic system; UPLC-MS, Ultra-performance liquid chromatography–mass spectrometry; UV, Ultraviolet; [C8mim]Cl, 1-Methyl-3-octylimidazolium chloride; [N1111]Cl, Tetramethylammonium chloride.
