# Supplementary material for: Beyond Composition: Structure–Activity Relationships in Bioactive Deep Eutectic Systems
Source: Pharmaceutics. 2026 Aug 11;18(8):990. doi: 10.3390/pharmaceutics18080990 (PMC13516601; doi:10.3390/pharmaceutics18080990)
Supplement: Supplementary file 1 [file pharmaceutics-18-00990-s001.zip › pharmaceutics-4437808-supplementary.pdf]

File S1. Search strategy and eligibility criteria

Parameter	Setting
**Databases**	PubMed; ScienceDirect
**Date range**	January 2016 – May 2026 (systematic database search); foundational pre-2016 works added through reference-list screening.
**Language**	English and Spanish
**Fields**	PubMed: title/abstract [tiab]. ScienceDirect: title, abstract, keywords
**Searchers**	Four authors, complementary topic domains; records pooled and de-duplicated by DOI
**Additional sourcing**	Reference-list screening of retrieved articles, including works published before 2016 where required for context.

Strand 1 — reported biological effects (PubMed syntax)

("deep eutectic"[tiab] OR NADES*[tiab] OR "low transition temperature mixture"[tiab] OR "low transition temperature mixtures"[tiab])

AND

(antimicrob*[tiab] OR antibacterial*[tiab] OR antibiofilm[tiab] OR antifungal[tiab] OR "anti-inflammatory"[tiab] OR inflammat*[tiab] OR immunomodulat*[tiab] OR cytokine*[tiab] OR interleukin*[tiab] OR "NF-kB"[tiab] OR "tumor necrosis factor"[tiab] OR "nitric oxide"[tiab] OR "oxidative stress"[tiab] OR cytoprotect*[tiab] OR regenerat*[tiab] OR "wound healing"[tiab] OR "tissue repair"[tiab] OR angiogen*[tiab] OR "cell migration"[tiab] OR "cell proliferation"[tiab] OR cryoprotect*[tiab] OR cryopreserv*[tiab] OR cytotox*[tiab] OR toxicit*[tiab] OR "cell viability"[tiab] OR IC50[tiab] OR apopto*[tiab] OR haemolysis[tiab] OR hemolysis[tiab] OR biocompatib*[tiab])

AND 2016/01/01:2026/05/31[dp]

AND (English[lang] OR Spanish[lang])

Strand 2 — physicochemical and structural basis (PubMed syntax)

("deep eutectic"[tiab] OR NADES*[tiab] OR "low transition temperature mixture"[tiab] OR "low transition temperature mixtures"[tiab])

AND

(nanostructur*[tiab] OR microstructur*[tiab] OR microheterogen*[tiab] OR "hydrogen bond"[tiab] OR "hydrogen bonding"[tiab] OR supramolecular[tiab] OR clustering[tiab] OR "water content"[tiab] OR hydration[tiab] OR "water activity"[tiab] OR viscosity[tiab] OR polarity[tiab] OR dielectric[tiab] OR "molecular dynamics"[tiab] OR "neutron scattering"[tiab])

AND 2016/01/01:2026/05/31[dp]

AND (English[lang] OR Spanish[lang])

ScienceDirect. The PubMed strings above were translated into ScienceDirect's Advanced Search using the same Boolean concept blocks, applied across the title, abstract and keywords fields; database-specific field syntax was adapted accordingly.

Eligibility

Criterion	Applied rule
**Agent**	A compositionally defined eutectic system (stated hydrogen-bond acceptor and donor, with molar ratio) is the entity under evaluation.
**Outcome, strand 1**	At least one biological outcome measured in a cellular, tissue or animal model: antimicrobial or antibiofilm activity; inflammatory, immunomodulatory or oxidative-stress readouts; cytoprotection, migration, proliferation, angiogenesis or wound closure; cryoprotection; viability, IC50, apoptosis or haemolysis.
**Outcome, strand 2**	Experimental or simulation data on structural or physicochemical properties, including spectroscopic, scattering and molecular dynamics studies.
**Not retained**	Eutectic system used only as an extraction or synthesis medium and removed before evaluation; effect attributable solely to a loaded active compound rather than to the system; system not compositionally characterized; acellular or analytical-method data only (strand 1); subject outside the biomedical field, as in bioremediation or ecological impact assessment; published in a language other than English or Spanish; full text unavailable.
**Screening**	Records were screened by title and abstract in Zotero®; the full texts of retained records were then read and assessed against the criteria above. Records whose eligibility was uncertain were discussed among the authors before a decision was reached. The final set was analysed and the evidence synthesized.
